# A qualitative study of the experiences of Syrian physicians in Türkiye: the need to strengthen integration

**DOI:** 10.1186/s12960-025-01023-1

**Published:** 2025-11-11

**Authors:** Abdulkader Mohammad, Diana Rayes, Sevgi Canbaz, Selma Karabey, Aula Abbara

**Affiliations:** 1 Faculty of Medicine, University of Novi Sad, Novi Sad, Serbia; 2 Syria Public Health Network, London, UK; 3 Georgetown University, Washington D.C., US; 4 Department Public Health, Istanbul Faculty of Medicine, Istanbul University, Istanbul, Türkiye; 5 Department of Infectious Diseases, Imperial College London, London, UK

## Abstract

**Introduction:**

Türkiye has been among the most welcoming for Syrian healthcare workers who have been forced from their homes due to the protracted conflict in Syria. It provides two main routes for work in Türkiye: one through formal accreditation processes to work in jobs equivalent to Turkish doctors and another through retraining as generalists to work in the Migrant Health Centres which cater to Syrian refugees across Türkiye. The aim of this study is to explore the experiences of Syrian physicians living or working in Türkiye.

**Methods:**

We used purposive and snowball sampling to identify Syrian physicians who were living or working in Türkiye at the time of the study. Interviews were conducted remotely in Arabic between February and August 2021 then transcribed and thematically analysed using a deductive approach to identify themes and subthemes.

**Results:**

Twenty three physicians were interviewed; 6 were female. Most resided in Gaziantep (15 of 23). The main themes are grouped as 1. Bureaucratic and academic challenges 2. Language, culture, and integration and 3. Personal impacts and future intentions. Many participants spoke of the prolonged journeys and challenges they faced whichever paths they chose; for many, obtaining ratifications of their certificates from the Syrian ministries was not possible, limiting their options. Themes around integration—related to language and culture (both in healthcare and in the community) also emerged.

**Discussion:**

Though Türkiye has provided the most favourable circumstances for Syrian physicians in the region, many personal and structural challenges remain; these impede successful career progress and integration. The interviews were conducted in 2021, during the COVID-19 pandemic. Since then, economic and political upheavals and natural disasters have affected perceptions towards Syrian refugees in Türkiye, including for physicians. In early December 2024, the fall of Syria’s regime will also likely have important impacts on physician intentions for remaining in Türkiye, return to Syria or migrate elsewhere. Further exploration of the impact of such changes is required to better understand the current experiences and intentions of Syrian physicians in Türkiye.

## Introduction

According to the official reports, Türkiye hosts around 3.6 million Syrians who have been forcibly displaced by its protracted conflict which escalated after uprisings were violently suppressed in March 2011; however, various national and international sources suggest that the unofficial figures range between 5 and 6 million. This discrepancy stems from estimates that include Syrians who are not registered under temporary protection as well as those holding different legal statuses in Turkey [1]. Their arrival increased from 2012 with many choosing to stay in Türkiye given its proximity to Syria (in the hope of return) and the presence of local and international humanitarian organisations in southeastern Türkiye for the cross-border humanitarian response to northwest Syria where many Syrians became employed [2, 3]. The latter encouraged healthcare professionals (HCPs) to remain in the area as it provided work opportunities and transit between Türkiye and Syria when border crossings were more fluid. The Turkish health system is a mixed one combining public and private financing. However, a significant part of this system is publicly financed and managed by the Ministry of Health. Due to the arrival of millions of immigrants in the country in a short time and the difficulties in using the existing health system, the Turkish Ministry of Health sought a solution to meet the primary healthcare needs of migrants. Thus, the Turkish Ministry of Health, with the funding support of the EU (European Union), has established the Migrant Health Centres through the SIHHAT project [4]. These centres not only provide an entry point to the national health system for refugees but also allowed Syrian doctors and nurses to retrain and work as generalists with limited registration in these centres; feedback from these centres has been positive with patients citing a breakdown of communication barriers compared to directly using the Turkish health system [4]. On the other hand, the problems that have emerged in the country's economy in recent years and high inflation have various negative reflections on the financing of health services.

The exact number of HCPs in Türkiye is unknown as not all are registered in Türkiye, as many work with humanitarian organisations in roles which do not require registration, some may work without formal registration, and some may reside in Türkiye but work inside Syria [5]. Despite insecurity and restrictions of travel between Türkiye and northwest Syria, for some, their ability to support the health system in northwest Syria, opportunities for work and remuneration without needing to undertake further exams or training and language barriers contributed to this decision [5]. For HCPs who remain in Türkiye, policies which support opportunities for work are among the more favourable for Syrian HCPs who are refugees than other countries in the eastern mediterranean region [6]. However, challenges to entering a new health system, particularly in a country where language is not shared contribute to a reluctance among Syrian HCPs to register to work in Türkiye or encourage them to look for opportunities elsewhere, particularly in Europe [7].

The aim of the study was to explore the experiences of Syrian physicians living or working in Türkiye to understand what facilitators or barriers exist for Syrian physicians. The study also aimed to expand understanding regarding the impact of the arrival of displaced Syrian HCPs on the Turkish health system more broadly, including what lessons can be learned from the experience of current Syrian HCPs in Türkiye. We focused on physicians due to the more streamlined processes for registration and work as well as the accessibility of key informants (KIs).

## Methods

We used a qualitative research method which included key informant interviews (KIIs) with Syrian physicians who are living or working in Türkiye; these include those working in the Turkish Health System or who are in the process of accreditation and registration. Transcribed texts were thematically analysed and classified into themes and sub-themes using a deductive approach.

### Recruitment

We used purposive then snowball sampling (the latter included recommendations from initial KIs who were identified purposively) to identify KIs using the inclusion and exclusion criteria below. Initial KIs were known to the authors (AA) from previous work with Syrian HCPs in Türkiye. Preliminary contact was made by AA or MA through WhatsApp (a preferred mode of communication among participants) with the objectives of the study. Those who showed initial interest were provided with a Participant Information Sheet (PIS) in English or Arabic as to their preference. Those who remained interested were able to ask any further questions before the interview. Once agreed, verbal informed consent was provided at the time of the interview.

### Inclusion and exclusion criteria

Inclusion criteria for eligibility included being aged 18 years and over and able to consent; being a Syrian physician currently living or working in Türkiye; available and willing to be interviewed by WhatsApp, Zoom or telephone during the study period. Exclusion criteria included not being a Syrian physician in Türkiye and not being able or willing to consent.

### Key informant interviews (KIIs)

KIIs were conducted remotely over WhatsApp or Zoom between February and August 2021 using a semi-structured interview guide (see appendix). These focused on the participants’ prior medical education and experience in Syria, the process of integration into the Turkish healthcare system, including the accreditation and/or adaptation procedures. The interview also explored the participant’s overall take on attitudes towards refugees in Türkiye, their personal health and well-being, and future plans as well as the impact of the COVID-19 pandemic on their integration process. Interviews were conducted in Syrian Arabic by AM who is a fluent Arabic speaker and a HCP of Syrian background. All interviews were recorded for transcription. All participants were anonymized (see table 1).

**Table 1:** Characteristics of the Study Participants and Durations of Interviews

Participants no	Age	Gender	Speciality	Interview duration (minutes)
01	31–40	Male	ENT	30
02	41–50	Male	Surgeon	55
03	41–50	Male	ENT	50
04	31–40	Male	General practitioner	40
05	31–40	Male	Surgeon	> 60
06	41–50	Male	Internal medicine	45
07	41–50	Male	Paediatrician	–
08	41–50	Male	Psychiatrist	> 60
09	41–50	Male	Surgeon	45
10	31–40	Male	Surgeon	45
11	31–40	Male	Surgeon	45
12	31–40	Male	Emergency medicine	30
13	31–40	Male	Ophthalmologist	40
14	31–40	Male	Paediatrician	30
15	41–50	Male	Surgeon	30
16	31–40	Male	Surgeon	30
17	31–40	Male	Surgeon	50
18	41–50	Female	Family medicine	> 60
19	31–40	Female	General Practitioner	50
20	41–50	Female	Obstetrics and gynaecology	30
21	41–50	Female	Obstetrics and gynaecology	40
22	51–60	Female	Paediatrician	40
23	41–50	Female	Surgeon	30

### Transcription and data management

Interviews were transcribed verbatim in Arabic by MA within 2–5 days of the interview being completed to ensure accuracy.

### Analysis

Interviews were read to familiarise with the content; the Arabic transcripts were uploaded for analysis by MA using Dedoose qualitative software. After a process of review and familiarisation, thematic analysis was then undertaken as per Braun and Clarke’s six-phase framework to identify recurring patterns, key themes, and subthemes related to the experiences of Syrian physicians in Türkiye [8]. A deductive coding approach was used to identify themes from existing literature and patterns within the themes covered in the interview guide, including experience of integration, prejudice and discrimination, impact on well-being and future plans. These codes were reviewed and discussed by another researcher (AA) to ensure consistency and comprehensiveness of analysis. This was then agreed with coauthors.

### Ethics

IRB was obtained from Istanbul University, Türkiye. All data were stored securely in password protected computers and files with only MA, AA and DR able to access them. KIs were assigned numbers so that their names and details were not visible except to the main researchers who were analysing the data. No KIs showed signs of distress during the interviews; however, if they had, interviews would have been terminated and support through a names counsellor provided. WhatsApp and Zoom were chosen with end-to-end encryption.

## Results

A total of 23 physicians participated in interviews of whom 6 were female. Fifteen resided in Gaziantep, while the remaining physicians were spread across Istanbul, Mersin, Izmir, and Ankara. All participants held Syrian nationality, and some also possessed Turkish citizenship. The mean age of the participants is 40.7 ± 5.8 (range 35–55). Most of the interviews, conducted exclusively via Zoom from February to August 2021, lasted approximately between 30–60 min each but three lasted longer than one hour. All sessions were recorded, numbered, transcribed, and subsequently analysed anonymously. Due to a technical problem, one of the recorded interviews (interview number 7) could not be incorporated into the project. Consequently, our analysis focused on the data from the remaining 22 interviews.

**Fig. 1:**
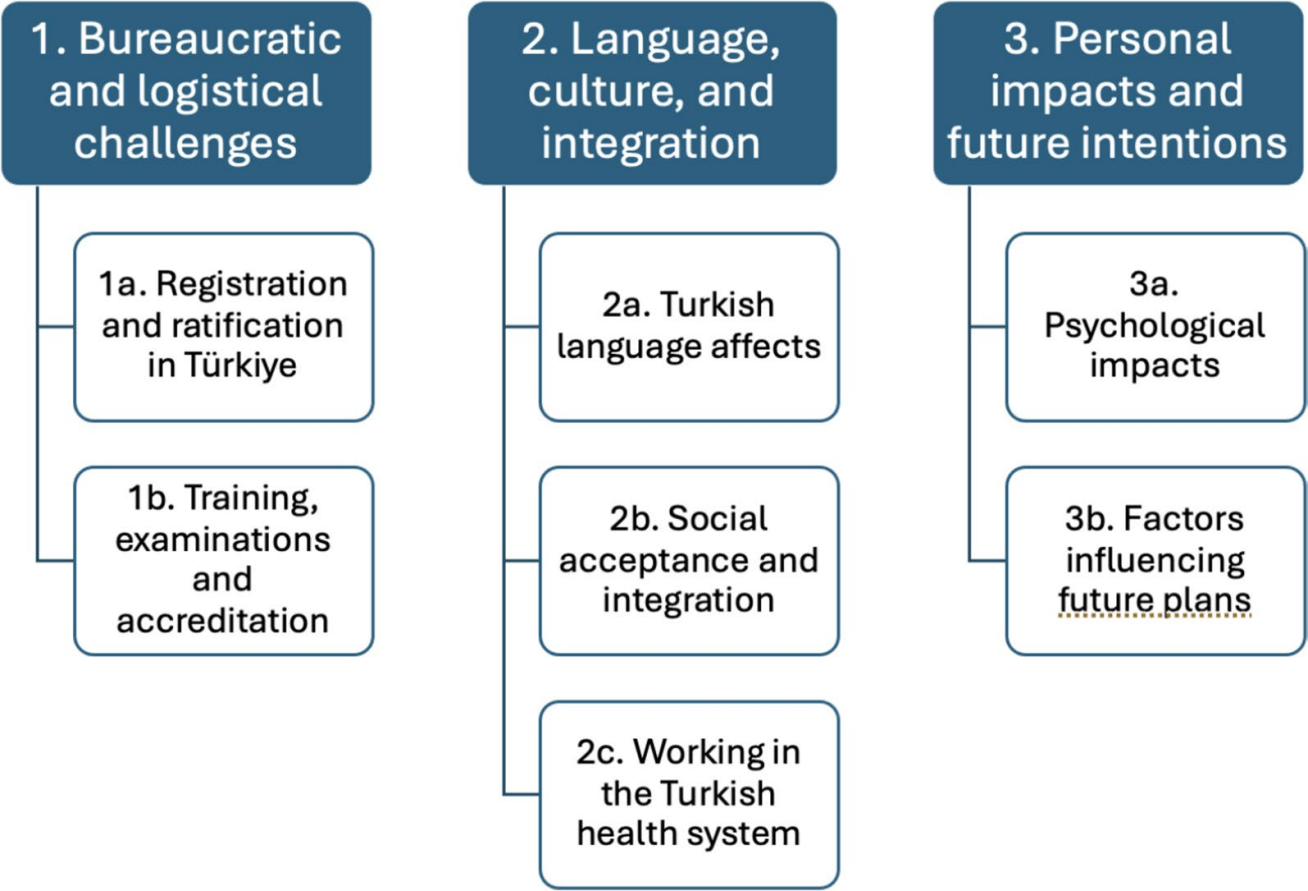
This figure shows the main themes and sub-themes

Three key themes emerged from the interviews. These were 1. Bureaucratic and logistical challenges 2. Language, culture and integration and 3. Personal impacts and future intentions. Related sub-themes are summarised in Fig. 1.

### Bureaucratic and academic challenges

All participants highlighted challenges around the registration process for working in the Turkish health system. This affected the ratification of their certificates, accreditation for previous training or specialisation and their ability to register in Türkiye.

#### Registration and ratification in Türkiye

Participants noted that for registration to work in Türkiye, the process was prolonged, arduous and could still mean that they would work as generalists or far from their families. They described a process where on arrival, they undertook theoretical and practical courses after which they underwent an interview to verify the authenticity of their certificates. They then undertook further practical experience and training including a few weeks in a Turkish hospital or clinic to get used to the Turkish system and learn to write prescriptions in Turkish. A participant explained:

“However, employment was not guaranteed for all doctors; it was determined by a lottery system. For example, on a particular day, there would be a lottery for paediatricians. Out of every 25 paediatricians, they selected two doctors to work in the clinic. Unfortunately, I was not lucky in several lotteries. Then they informed us that if we wanted employment, it would not be as paediatric specialists but as general practitioners. Many doctors did not agree.” (no.22)

Some took the option of working as generalists in Migrant Health Centres given the many hurdles which existed for specialist registration. For the recognition of specialisation, this required the validation of documents from both inside Syria and the Syrian embassy in Türkiye. A participant noted:

“… but for the specialisation, we cannot fulfil this, because they ask for ratification from the Syrian Foreign Ministry and the consulate, and we cannot do it, and it is impossible to return to Syria to obtain it, and the consulate does not certify any certificate, and this is a dilemma for the recognition of the specialisation.” (no.20)

Another participant described the bureaucracy saying:

“Do you remember the bureaucracy in Syria? Of course, corruption and bribery are a very bad thing, but the bureaucracy in Syria could be overcome with 500 Syrian pounds, by imposition, by gift …. but here you cannot jump over these barriers, the barriers are doubled here.” (no.19)

#### Training, examinations and accreditation

Participants noted that the need to take formal examinations, often involving basic science for reaccreditation, could be very challenging especially while working alongside or for those who had graduated many years before. One participant explained:

“It is not reasonable to make a specialist doctor repeats the entire human medicine exam when they completed their university degree 15 or 20 years ago, or even more studying. For example, an internal specialist should take an internal medicine exam, a paediatrician should take a paediatric exam, and a gynaecologist should take a gynaecologists’ exam.” (No.16)

### Language, culture and integration

Participants highlighted the challenges for both social integration and integration in the Turkish health system which related to language barriers and cultural differences. Challenges related to learning Turkish, something which is essential to passing required examinations or undertaking clinical observations in Turkish hospitals, was seen to be a barrier to more rapid progression towards registration. Those who were fluent in Turkish reported feeling more able to socialise with Turkish colleagues and work more easily in the health system.

#### Turkish language

Most participants noted the challenge around language barriers which affected their accreditations, success at examinations as well as integration in Turkish society. One participant noted:

“Language is the first obstacle so far; Syrians have not mixed with Turkish society. They tend to congregate in specific areas, so a person doesn't have to use a language other than Arabic in their daily tasks”. (No.13).

Another participant reflected on personal language challenges, saying:

“The language challenges have become easier for me recently on a personal level. However, in my previous experience with the equivalency exam, I couldn't pass the test due to misunderstandings of the question or not understanding of certain words.” (No.02).

Others noted the limited availability of Turkish language courses, unlike in Europe where these were encouraged and more readily available. They noted:

“Regarding the language issue, there are no facilitators or educational courses like those provided by European governments”. (No.03)

#### Social acceptance and integration

The participants described a wide range of experiences in their interactions with Turkish society. Some reported positive experiences with support and advice while others reported discrimination and a lack of acceptance which affected them psychologically and affected their children. This included variable experiences towards integration. One summarised it as:

“The higher the pressure and the lower the relief, the greater the susceptibility to mental illness. Surely, those who live in difficult financial circumstances, like me who lost everything in my country, are also subjected to discrimination. All of this has an impact on a person's psyche and emotions, and it may give rise to resentments that are not easy.” (No.8)

Some expressed regret at staying in Türkiye rather than moving elsewhere. One said:

“There are many doctors, and I may be one of them. I regret that I stayed in Türkiye, although I could have left Türkiye for Europe or the Gulf, but I chose Türkiye, and we did not expect it to take your time and life without any return.” (No.23)

Others noted the psychological toll on their children who faced discrimination. A participant said:

*“When my children first entered Turkish schools, they were beaten, and one had his hand broken and his glasses were broken. My children are polite and not of the type [to get into fights] but I can do nothing. When I went to the school, they said what should we do; all the children are fighting, and your children are very polite…*.”

Others described more favourable interactions and spoke of how the customs and culture in Türkiye are similar to Syria and that overall, the situation in Türkiye was more favourable to them than Europe. A participant noted:

“On the contrary, I chose Türkiye despite my completion of the German language for the sake of my children, my family, and the culture and the proximity of the country. So, I preferred to stay in Türkiye over other countries, despite some of the difficulties in Türkiye relative to other countries in Europe.” (no.10)

#### Working in the Turkish health system

Most participants did not work in the Turkish health system noting that it was challenging to secure employment even in the Migrant Health Centres. Others noted that the pathway to working either in the Turkish health system or in the Migrant Health Centres was difficult, with little support, leading them to choose either to work in non-governmental organisations or unofficially as doctors. Participants noted:

“As for the newcomers, there are no courses, and their situation is worse as they do not have an opportunity to work. Some of them found an opportunity to work with [humanitarian] organisations and now they work but the rest are currently without work”. (no.16)

Another suggested:

“Opening a hospital for Syrian specialists in Türkiye will make it easier for the Syrian patients and relieve pressure on Turkish hospitals; there are many graduate and specialised doctors who do not have job opportunities.” (no.13)

For those with experience of the healthcare system in Türkiye either through work or as patients spoke well of the electronic medical records and health information systems which were not commonly used in Syria. However, they noted differences in clinical practice which included that Turkish doctors relied more on investigations rather than clinical examinations, unlike how Syrian doctors practise. Participants noted:

“When we received the patient, we dealt with him with our hands and diagnosed them, but here they do not do that. Once the patient enters, the prescription is written without using a stethoscope or tongue depressor for diagnosis. Here they rely on analysis and radiographs for diagnosis. On the contrary, in Syria we rely on diagnosis from personal experience.” (no.22)

However, another participant acknowledged that this difference in practice was due to the presence of more available resources. They note:

“Due to the availability of medical resources and equipment in Türkiye, a number of investigations are requested, which may be beneficial for the patient and reduces the time to reach a diagnosis. This is unlike the situation in Syria, where an MRI scan is not requested until some time has passed.” (no.06)

### Personal impacts and future intentions

The perceived challenges explored had a profound psychological impact that was additional to the strains which related to the COVID-19 pandemic, the displacement they had experienced and the impact of the ongoing armed conflict in Syria. They noted the impact of increased pressures which also related to often needing to juggle work while also learning Turkish or revising for exams and trying to maintain a balance between work and their families. The increasing challenges which they faced which included an increase in perceived racism or bias in the system also influenced their future plans.

#### Psychological factors

Most of the participants described incidents of racism, a feeling of non-acceptance, and a lack of respect that affected them in Türkiye. They reported:

“During the training period in Gaziantep, and during the first training day, all of our names were documented but we were prevented from attending [clinical training]. We spent the training period (three months) in the hospital garden.” (no.9)

For participants who were older, they described a lack of respect among more junior staff while working. A participant noted:

“Resident doctors are students to us, and we are the age of their teachers, yet they were controlling us and this is something we cannot forget.” (no.21)

Others noted incidents where they were treated well and other times not. One gave the example:

“It was clear to me that there is a professor who invites you to his office, welcomes you and tells you that ‘we are brothers’. Another professor prohibits you from speaking in English and compels you to speak Turkish, relishing in the fact that you appear not to understand.” (no.8)

#### Factors influencing future plans

All the participants described the main factors which affected their future decisions to stay or leave Türkiye; these included economic factors as well as ongoing insecurity in Syria. The proximity to Syria in case of the possibility of return was discussed by more than one participant. They also noted:

“I am obliged to continue here as I have significant expenses. I currently have two children in university, and the three thousand Turkish liras [I earn] are not sufficient for their university costs, including rent and other expenses. The second child is currently taking YOS [Turkish Foreign Student Examination] exams, and if he does not pass, he will need to retake the exam. Additionally, I have three daughters in private schools, and two of them require special care due to health issues. Consequently, I must remain and work as I do not have enough to cover my monthly expenses, which total 25 thousand Turkish liras.” (no.21)

Many participants indicated their intention to return to Syria however the protracted conflict and ongoing insecurity as well as the lack of change in the government makes this less feasible as an option leading some to reconsider. This is particularly so due to concerns about the security and safety of their families as well as the need for educational opportunities for their children. Participants said:

“If the [Syrian] regime changes, of course, we will return. If Assad is removed, we will all return. But if he remains, how will we return? Impossible.” (no.20)

“The survival of the political system is another factor that I cannot return to Syria unless it changes; I can return only if the political system is removed. I promise you that most of the doctors in different countries will return.” (no.1)

With regards to safety and security, one participant remarked:

“The lack of complete security will prevent me from settling down with my family in Syria. Entry and exit from Syria should be easy so that I can return to my family. Security is important because I am the sole provider for my family, and any danger to me would cause suffering for my family.” (no.02)

## Discussion

Though Türkiye has among the most favourable policies for Syrian HCPs to retrain or be integrated into the Turkish health system, Syrian physicians faced many challenges both on a personal and professional level. What marks Türkiye out compared to other countries which host Syrian refugee physicians is the possibility of retraining as generalists to work in Migrant Health Centres. Research with Syrian refugees find that this improves healthcare access to primary care [4] but for Syrian physicians, working in these centres can present frustrations, particularly for those who are more senior and who are already specialised as seen in our study. Our main themes note the bureaucratic challenges, those which relate language, culture and integration as well as the personal impacts on the physicians and their families. In terms of potential return to Syria, at the time of the interviews, participants noted that they would consider this if there was a change of regime and the presence of security for them and their families. The fall of Syria’s regime in December 2024 may change the perceptions around return for Syrian HCPs, however, at the time of writing, its impact remains uncertain [9, 10].

### Pathways to working in Türkiye

**Fig. 2:**
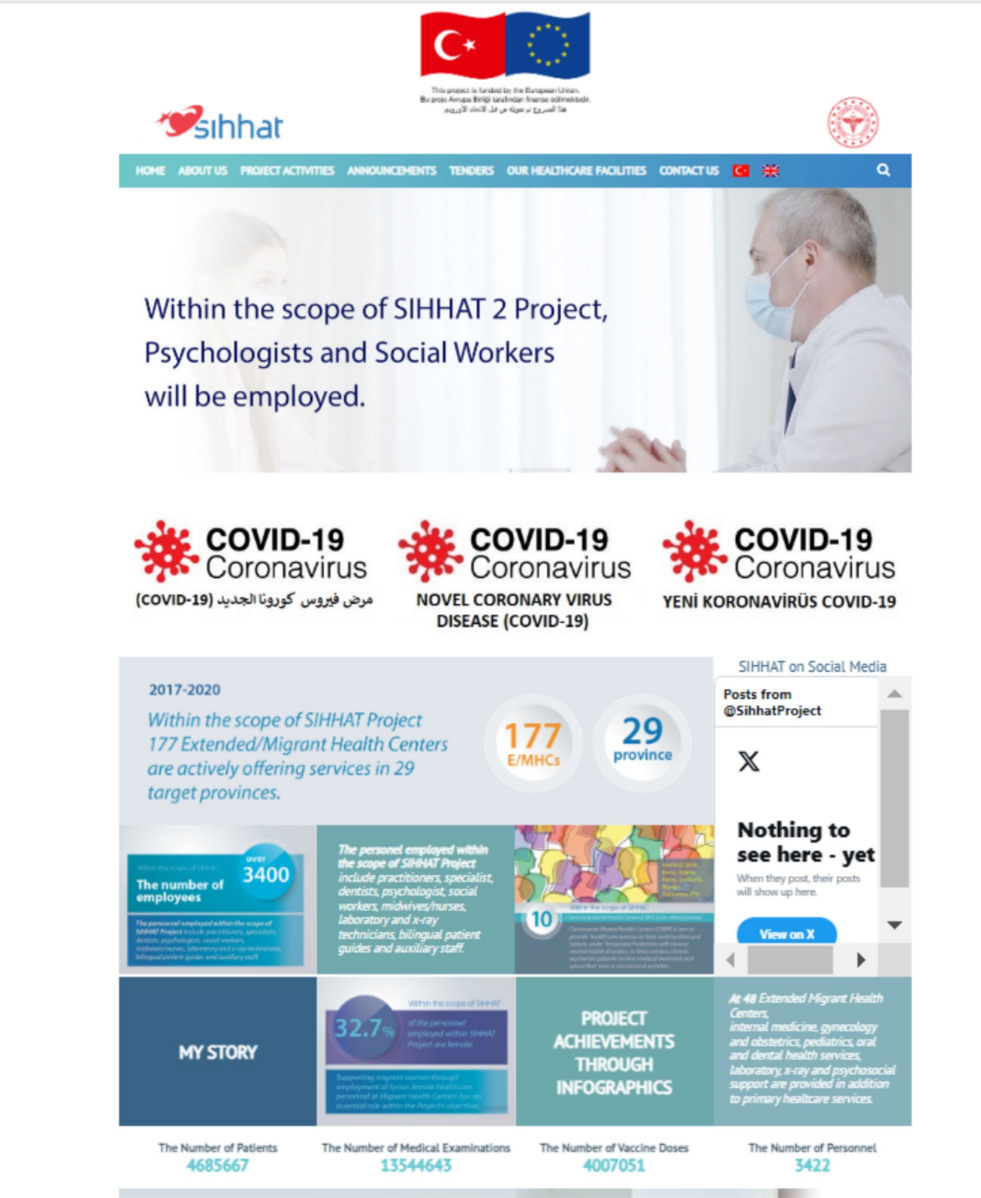
The website of the Health Project, which operates migrant health centres [14]

Türkiye hosts the largest number of Syrian refugees globally and has been among the most welcoming including opportunities for HCPs to work despite the challenges which Syrian physicians describe. Notably, in other regional countries which host Syrian refugees including Lebanon (780,000 registered and more unregistered) and Jordan (630,000) restrictions have been placed on the ability of Syrian physicians and other HCPs to work [11]. As a result, some work in the informal health sector leaving them at risk of exploitation, legal consequences or repatriation to Syria, something which is unsafe for many Syrian HCPs [12, 13]. As such, despite the challenges for physicians to work, many report being grateful that such opportunities exist. Importantly, though the presence of Migrant Health Centres in Türkiye represents the establishment of a parallel health system for primary care services (something which advocates of the integration of refugee into local health systems advise against,) this provides the opportunity for physicians to work. Syrian patients in Migrant Health Centres also report a high satisfaction with the services due to the ability to Syrian physicians who can communicate with them and understand their cultures [4]. More details are found on the SIHHAT project website and in Fig. 2.

The inclusion or integration of physicians who are refugees into host health systems can be challenging; this is mainly done with the expectation that the refugee physicians undertake language examinations, ratification of their certificates and examinations in the same way that other migrant physicians would [15]. What our KIs describe, holds some similarity with some of the challenges noted in other countries e.g. language, duration to accredit while other challenges are Türkiye specific e.g. conditional registration to only work in Migrant Health Centres [10].

In Europe, Germany is the country which hosts the largest number of Syrian refugees; data from 2024 notes that 6,000 Syrian physicians are registered, accounting for 10% of the international doctors who are registered in Germany [16]. A 2019 article noted that despite the streamlined process of registration which is in place, Syrian physicians faced many challenges during the process [17]. This included bureaucratic challenges as well as challenges with language, medical culture and the availability and ratification of their certificates [17]. In the UK, though there have also been positive attempts to support refugee physicians (both Syrian and non-Syrian) to work in the NHS with investment from NHS workforce (formerly Health Education England, [18]) Despite this, refugee physicians face challenges in gaining employment in the UK’s NHS (National Health System,) even when they have obtained GMC (General Medical Council) registration, a situation which is similar that faced by migrant physicians to the UK. What marks the UK out from other Syrian refugee hosting countries is the third sector support directed towards refugee physicians; this includes support with clinical experience, accreditation and registration processes [19]. A narrative review by Farnham et al. examined the challenges which refugee health workers face in the UK which included trauma experienced, gaps in working lives, financial difficulties and legal challenges [20].

The concept of transitioning from a ‘respected’ physician with social status in Syria to another country where they needed to re-establish themselves also impacts feelings of self-esteem. A study of Syrian physicians in the Netherlands explores the pathways to accreditation as well as other concepts around social capital, symbolic capital, economic capital and cultural capital to explore the wider impacts on the Syrian physicians in their study [21]. They note that the practical elements of accreditation and registration in the Netherlands is challenging and closely intertwined with the broader social factors which impact integration and professionalism as perceived by the physicians. We did not explore such factors in-depth in our research, however, some elements around the loss of status, economic pressures and challenges around integration emerged.

Some KIs, particularly those who are already specialised or older and who wanted to work in their speciality rather than as generalists voiced frustration at the challenges that existed in being able to do this. The option of working as generalists in Migrant Health Centres was also less favoured due to lower salaries compared to Turkish physicians working the main health system. Not having formal specialist registration also disallowed for private practice to supplement their salary. For this reason, among others, some physicians favoured basing their family in Türkiye while they travelled into northwest Syria to work. This also allowed them to feel that they were supporting patients and the health system in parts of Syria which they could access.

### Challenges to integration

Beyond the logistical or bureaucratic challenges to undertake registration to work in Türkiye KIs reported challenges relating to socio-cultural acceptance and integration both in the course of their work but also socially. They spoke of differences in how they were treated compared to peers with sometime discriminatory practices. The challenges which refugee or migrant physicians face towards social acceptance whether in the workplace or social is something which has been documented in other contexts. For example, a study in Germany exploring the challenges and advantages in the integration of migrant physicians showed that migrant physicians may face integration challenges due to factors like limited language skills and difficulties in interacting with patients and colleagues [22]. Similarly, in Canada, migrant physicians face challenges not only in obtaining licensure and acceptance but also communication difficulties which could impact integration [10] [23].

Some KIs noted differences in clinical approaches, particular to investigations; this likely reflects differences in the health systems between Syria and Türkiye which existed before the conflict and since [24]. Different approaches to medicine are evident in different countries depending on the availability and cost of investigations in different health systems. In Syria, after the conflict, MRI scans are very limited or expensive in a system where most of these investigations will be at the patients’ own expense [25]. In a country where more than 90% live in poverty, this is out of reach for the majority [26]. Türkiye’s health system, on the other hand, provides more comprehensive coverage for diagnostic tests, including MRI and other expensive tools, through universal health coverage. This system gives more options for doctors compared to Syria and reduces a large portion of healthcare costs for patients. Additionally, Türkiye has a large number of public hospitals equipped with advanced medical diagnostic tools, which allows for greater access to care, especially in rural areas, compared to Syria.

Beyond integration or acceptance in the workplace, some reported facing challenges to social integration however, KIs noted that fluency in Turkish positively influenced such integration. Such challenges for refugee physicians are not unique to Türkiye. In Germany bureaucratic hurdles and long wait times for credential recognition impact inclusion into the health system with consequences for both professional as well as social integration [17]. Additionally, language barriers and cultural differences can make it difficult for them to integrate and build connections in the community.

### Impacts and future intentions

Syrian physicians had wanted to stay in Türkiye for a number of reasons including the proximity to Syria (with some taking the option of living in Türkiye but working in Syria) as well as the initially positive welcome and support received. However, the worsening socioeconomic situation in Türkiye and the changing political situation at the time of the interviews but also after COVID-19 and the earthquakes may have altered the opportunities available to Syrian physicians and their intention to remain or move elsewhere. The earthquakes had affected areas in southeast Türkiye where a large proportion of Syrian refugees live; as such, the homelessness and loss of opportunities that occurred in its aftermath would also have impacted attitudes. Some of the stated intentions by the interviewees about whether they remain in Türkiye, return to Syria or move elsewhere related to their family responsibilities, the ongoing political instability in Syria and the challenges they and their families face in Türkiye. The December 2024 fall of Syria’s regime will also likely have important impacts on HCPs intentions for remaining in Türkiye, return to Syria or migration elsewhere however, its full impact remains uncertain at the time of writing.

In a review of policies around the return of healthcare workers to post-conflict health systems, security and opportunities for employment and livelihoods were key factors noted as barriers to return [27]. Other policies which may support return in a post-conflict phase include opportunities for health system rebuilding including education and training but also support areas of greatest need e.g. rural areas on their return. One example is of Sudanese refugees (who had trained in Cuba) who returned from Canada to South Sudan to work in rural areas [28]; they benefited from a training program established by the University of Calgary and Samaritan’s Purse as a means of supporting a ‘reverse brain-drain’ approach. For Syrians, the situation for returnees remains high risk particularly in areas under government control which could lead to a reluctance for voluntary return [29]. Alongside this, opportunities for onward migration to Europe are becoming increasing dangerous and restrictive, including to countries with high numbers of Syrian refugees including Germany or the Gulf Countries, even for physicians who had been in high demand [30].

### Strengths and limitations

To our knowledge, this is the first qualitative research study which explores the situation for Syrian physicians in Türkiye and highlights some important issues about the challenges they face. Despite this, there are limitations. These interviews were conducted in 2021, soon after the COVID-19 pandemic started; this may have influenced the findings given the situation at that time but also the more recent changes which have occurred in both Türkiye and Syria since then, including after the February 2023 earthquakes. However, the fall of the regime in early December 2024 is likely to have important impacts on the experiences and intentions of Syrian physicians in Türkiye which need to be further understood. The interviewees are physicians who, in many refugee hosting countries often have greater opportunities to retrain and registered compared to other cadres; as such, their views cannot be extrapolated to the situation for other HCPs who may face some similar and some different challenges. As such, further interviews which focus on future intentions and factors which may support safe return to Syria as well as interviews with non-physicians are required.

## Conclusions

We find that though Türkiye has by far the most favourable support for the registration and employment, Syrian physicians report various challenges to employment and integration. These interviews were conducted during 2021 and, as such further interviews with physicians and non-physicians are important to understand how the situation has evolved as this will provide useful information for future health workforce planning in Syria. The fall of Syria’s regime in early December 2024 is likely to have important impacts on the experiences and intentions of Syrian physicians in Türkiye which require further understanding. As such, further exploration of the impact of such changes is required to better understand the experiences and intentions of Syrian physicians in Türkiye.

## Data Availability

Data is provided within the manuscript or supplementary information files. Further data from the transcribed interviews can be made available on a case by case basis on request from the corresponding author but no data that could adversely affect participants will be made available.

## Appendix

Interview guide:

## Semi-structured interview guide

Experiences of Syrian Healthcare Workers in Turkey

## Introduction

Thank you for agreeing to participate in this study. This research aims to explore the experiences of Syrian healthcare workers in Turkey to understand what facilitators and barriers exist for Syrian healthcare workers studying or working in Turkey. This is with the aim of understanding the impact of the Syrian healthcare workforce on the Turkish health system, including what lessons can be learned from the experience of current Syrian healthcare workers in Turkey.

This interview guide will cover questions which include your prior education and work experience in Syria, as well as your current experience in Turkey. The guide will touch on topics relating to your continued training or adaptation of your degree, as well as your personal and professional experiences inside Turkey.

## A. Prior experience in Syria

- Tell me about your background.
- What is your profession/specialty?
- Where did you complete your education/training?
- Tell me about your work experience.
- Where have you worked?
  o Inside or outside of Syria?
- How long have you worked in each setting?
- Have you worked in more than one state in Turkey?

## B. Current experience in Turkey

1. Please explain your experience of integration into the Turkish healthcare system.
  o How did you obtain proof of degree and accreditation?
  o What are some of the challenges or difficulties ratifying Syrian diplomas?
  o Have you experienced any challenges related to bureaucracy in Turkey?
  o How much time did it take/is it taking for full registration?
  o What are the financial costs of exams and living expenses in Turkey?
  o Other (i.e. Despite programs in place to facilitate integration, what are the remaining challenges?)
2. Please explain your experience re-training or adapting your degree.
  o Did you experience any long delays in practice due to conflict/displacement?
  o Did you face any barriers due to culture and language? Please provide examples.
  o What are some of the differences in clinical practice in Turkey and how were they overcome?
  o Please describe potential political influences on your ability to adapt your degree.
  o What are your long-term career plans (return to Syria/ stay in Turkey)?
3. Attitudes towards refugees in Turkey
  o Please describe the level of acceptance (or lack thereof) you feel from Turkish colleagues and patients.
  o Have you noticed any changes in attitudes towards refugees in Turkey? Please provide examples.
4. Personal health of healthcare workers
  o [If no longer a refugee] Please describe your experience as a resident HCW in Turkey. How does this contrast with your experience as a refugee/migrant HCW?
  o Have you experienced any challenges integrating into Turkish society? Please provide examples.
  o What have been some of the mental health impacts of your integration process?
  o What are some of your personal values? Have they been fulfilled since you came to Turkey?
  o Is it important that you can work in Turkey? Why is that important to you?
5. **Impact of COVID-19**
  o If you have not already, please provide details on the impact of the COVID-19 pandemic on your integration process, including, adapting your degree, completing or starting training, attitudes towards refugees, personal health, and plans for the future.

## C. Future plans

1. If you are comfortable answering this question, what are your future plans to continue working in Türkiye /Syria?
2. Do you imagine returning to Syria in the near future? Please provide details on what personal and professional circumstances would need to be in place for you to consider return.

## D. Conclusion

1. Is there anything else you would like to add on this topic?
2. Do you know anyone else who would be willing to be contacted for an interview? (Particularly a HCW with Syrian/Turkish citizenship)
3. May we contact you again for a follow-up interview?
4. Do you have any questions for me?

Thank you.

## Abbreviations

ENT: Ear nose and throat
EU: European Union
GMC: General medical council
HCPs: Healthcare professionals
KIs: Key informants
KIIs: Key informant interviews
MHCs: Migrant health centres
MRI: Magnetic resonance imaging
NHS: National health service
PIS: Patient information sheet
UK: United Kingdom
